# Removal of an intraorbital metallic foreign body following double-penetrating ocular injury: A case report

**DOI:** 10.1097/MD.0000000000013790

**Published:** 2018-12-21

**Authors:** Yan Cui, Ziwei Li, Yuwei Wang, Long Shi

**Affiliations:** a Department of Ophthalmology, Qilu Hospital; b Department of Medicinal Chemistry, Key Laboratory of Chemical Biology (Ministry of Education), School of Pharmaceutical Sciences, Shandong University, Jinan, Shandong, China.

**Keywords:** extraocular muscle severance, intraorbital metallic foreign body, penetrating eye injury, transconjunctival approach

## Abstract

**Rationale:**

Open eye injury is one of the commonest ophthalmic emergencies, and when accompanied by intraorbital foreign bodies, the condition carries a poor prognosis.

**Patient concerns:**

A 28-year-old man presented to the emergency department of our hospital complaining of sudden painful loss of vision in the left eye after he hammered an iron plate.

**Diagnosis:**

The ocular examination revealed a 4-mm full thickness scleral laceration with prolapsed uveal tissue, a traumatic cataract. Computed tomography (CT) demonstrated an orbital foreign body in the retrobulbar area.

**Interventions:**

The patient underwent emergency scleral suturing, severance of medial rectus muscle, and removal of the orbital foreign body. Twelve days after the emergency operation, pars plana lensectomy and pars plana vitrectomy were performed.

**Outcomes:**

After 3 months of follow-up, there was no immune response. Visual acuity in the left eye was the perception of hand motion. The retina remained mostly attached with normal intraocular pressure, and good cosmetic appearance. The globe anatomy was maintained, but the vision could not be restored due to the grave nature of the trauma.

**Lessons:**

Transconjunctival approach extraocular muscle severance may thus be a suitable approach to the removal of intraorbital metallic foreign body.

## 1 Introduction

Penetrating eye injury is one of the commonest ophthalmic emergencies.^[1]^ The presence of intraorbital foreign body has been rarely associated with penetrating eye injury.^[2]^ When the injury is accompanied by intraorbital foreign body, the condition carries a poor prognosis. The intraorbital foreign body may cause damage in 2 different ways.^[3]^ It may produce structural damage to the intraocular contents, as it penetrates the eye or may cause toxicity to tissues, as it degrades and oxidizes, if not removed early.

In cases of penetrating eye injury by a foreign body that reaches the retrobulbar area, removal of the foreign body is generally complicated. In removing intraorbital foreign bodies, accurate localization of the foreign body and the choice of a safe and appropriate surgical procedure are vital. Available options for the removal of such an object include lateral orbitotomy and transcranial and transnasal approaches,^[4]^ while a transconjunctival approach is a simpler method and leaves no visible scar. We now report the successful removal of an intraorbital metallic foreign body by the transconjunctival approach with extraocular muscle severance.

## 2 Case description

**Figure 1:**
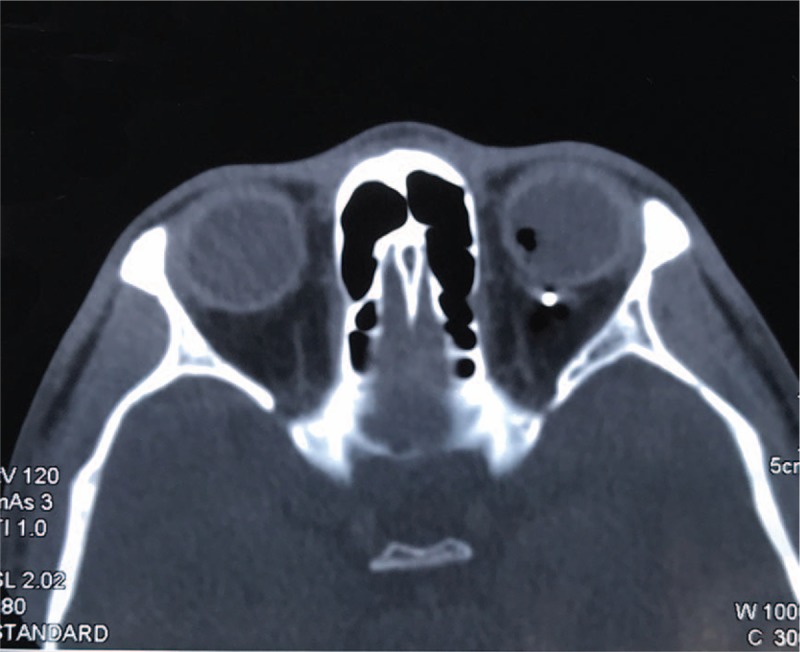
Orbital CT of a 28-year-old man with a double-penetrating ocular injury. Orbital CT scan revealed a high-density foreign body in the posterior region of the left retrobulbar space.

A 28-year-old man presented to the emergency department of our hospital complaining of sudden painful loss of vision in the left eye. He gave a history of hit by a sharp metallic object on the left eye while hammering an iron plate. His left eyelids were mildly swollen, but the orbital rim was intact with no crepitation. Visual acuity in the left eye was limited to the perception of hand motion, while the visual acuity in the right eye was 20/20, and the left intraocular pressure (IOP) was not measurable. Slitlamp examination revealed a full thickness scleral laceration of 4.0 mm length with prolapsed uveal tissue, a shallow anterior chamber, and a traumatic cataract. The details of the posterior segment could not be visualized. Computed tomography (CT) demonstrated an intraorbital foreign body with intensity of iron that had passed through the left eyeball and was located in the intraorbital space close to the optic nerve (Fig. 1). The right eye was normal.

This study was conducted at the Shandong University Qilu Hospital and the procedures used were approved by the Ethics Committee of the Shandong University Qilu Hospital. The procedures conformed to the tenets of the Declaration of Helsinki.

**Figure 2:**
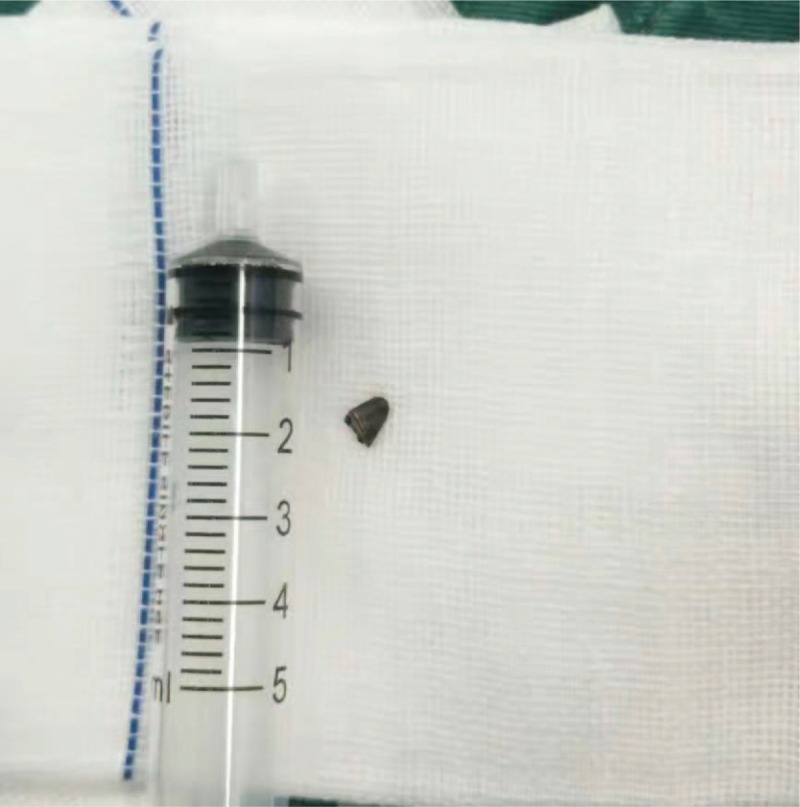
Removal of metallic foreign body. The metallic foreign body was removed through transconjunctival approach.

The primary repair of the scleral perforation with abscission of the prolapsed and necrosed uveal tissue was done on the emergency basis. The conjunctiva was incised along the limbus cornea, the sclera was exposed, and the scleral laceration was confirmed and sutured. We actually attempted to maneuver the foreign body behind the eyeball with the use of a magnet, but this was not successful. We therefore severed medial rectus muscle. An iron foreign body was found and was removed in a single piece (Fig. 2). From outside of the eye, the exit laceration could not be confirmed. The operation was completed without a scleral suture of the exit laceration. Postoperatively, the intravenous antibiotics were administered, topical antibiotics and steroids with cycloplegics. Visual acuity in the left eye was perception of hand motion, the wound was healthy with intact sutures, the anterior chamber was formed, and the lens was cataractous with no view of the retina. The IOP in the left eye was 13 mm Hg. Removal of the foreign body was confirmed by a postoperative CT scan.

Twelve days later, the patient underwent pars plana lensectomy and 3 ports (23-gauge) pars plana vitrectomy. We observed a scleral exit laceration near the optic disc, but did not see any objects. The full-thickness posterior pole defect was closed. With endolaser photocoagulation and silicone oil injection, the patient had his retina reattached. On follow-up after 4 weeks, the visual acuity was always limited to hand motion perception, scleral sutures were intact. The anterior chamber was formed. The IOP was 15 mm Hg. At 3 months after the operation, visual acuity in the left eye was the perception of hand motion and the left IOP was 15 mm Hg (noncontact tonometer). There were no postoperative complications (including retinal detachment, proliferative vitreoretinopathy, infection, sympathetic ophthalmia, and hemorrhage).

## 3 Discussion

We present a rare case that a metallic foreign body passed through the eye and located in the intraorbital space. The prognosis of the case is poor, but timely removal of the foreign body is essential to prevent serious complications.

Worldwide, open eye injury is an important cause of eye morbidity.^[5]^ The incidence of open eye injury is about 3.6 to 3.8 per 100,000 populations annually.^[6]^ If a foreign body causes the entrance and exit wound, it is regarded as a perforating injury or double-penetrating ocular injury. There is a strong preponderance that most patients with open eye injuries were young men. This reflects the aggressive characteristics of male behavior.^[7]^

The removal of an intraorbital foreign body is complicated. Due to associated high risk of orbital inflammation and serious complications, metallic foreign body should be removed. Metallic foreign body can be easily detected with orbital CT. Selection of the most appropriate approach remains controversial. Location, size, chemical structure, and the complications caused by foreign body influence the decision to remove it.^[8]^ Transcranial approach was used in removing an intraorbital foreign body,^[9]^ but this calls for neurosurgical competence and increases risk of intracranial complications. Lateral orbitotomy does not afford a good operating field of intraorbital foreign body; it might apply pressure to eyeball when attempting to remove foreign body. In our case, we performed removal of foreign body using a transconjunctival approach. The conjunctiva was incised along limbus cornea. Severance of medial rectus muscles to broaden the operating field allowed us to remove foreign body with minimal invasiveness. The surgical result was encouraging. The patient had a good cosmetic appearance, normal IOP, and maintained visual function. Hence, the transconjunctival approach is simpler and is a more feasible and effective method for removal of intraorbital foreign body.

The goal of penetrating ocular injury is to maintain function and integrity of eye. To achieve the goal, it is very important to perform a waterproof closure of both anterior and posterior penetration, in order to maintain a normal IOP. In case of open eye injury with small posterior scleral exit laceration, the exit laceration could seal itself and additional suture is not needed. In the animal model, small posterior scleral wounds were closed with fibrosis after injury.^[10]^ Aggressive manipulation could cause further hemorrhage and uvea prolapse. Our patient had a double-penetrating injury of eyeball; we were unable to suture the posterior wound. It sealed itself and there was no complication of retina.

We conclude that the transconjunctival approach and extraocular muscle severance can be considered as an appropriate method for removing intraorbital foreign body, because it results in fewer surgical complications and less orbital tissue damage.

## Author contributions

**Methodology:** Yan Cui.

**Writing – original draft:** Ziwei Li, Yuwei Wang.

**Writing – review & editing:** Yan Cui, Long Shi.
